# Current Review of Increasing Animal Health Threat of Per- and Polyfluoroalkyl Substances (PFAS): Harms, Limitations, and Alternatives to Manage Their Toxicity

**DOI:** 10.3390/ijms241411707

**Published:** 2023-07-20

**Authors:** Alessio Filippo Peritore, Enrico Gugliandolo, Salvatore Cuzzocrea, Rosalia Crupi, Domenico Britti

**Affiliations:** 1 Department of Veterinary Science, University of Messina, 98166 Messina, Italy; egugliandolo@unime.it (E.G.); rcrupi@unime.it (R.C.); 2 Department of Chemical, Biological, Pharmaceutical and Environmental Science, University of Messina, 98166 Messina, Italy; salvator@unime.it; 3 Department of Pharmacological and Physiological Science, School of Medicine, Saint Louis University, Saint Louis, MO 63103, USA; 4 Department of Health Sciences, Campus Universitario “Salvatore Venuta” Viale Europa, “Magna Græcia University” of Catanzaro, 88100 Catanzaro, Italy; britti@unicz.it

**Keywords:** PFAS, animal toxicity, environmental contaminant

## Abstract

Perfluorinated and polyfluorinated alkyl substances (PFAS), more than 4700 in number, are a group of widely used man-made chemicals that accumulate in living things and the environment over time. They are known as “forever chemicals” because they are extremely persistent in our environment and body. Because PFAS have been widely used for many decades, their presence is evident globally, and their persistence and potential toxicity create concern for animals, humans and environmental health. They can have multiple adverse health effects, such as liver damage, thyroid disease, obesity, fertility problems, and cancer. The most significant source of living exposure to PFAS is dietary intake (food and water), but given massive industrial and domestic use, these substances are now punctually present not only domestically but also in the outdoor environment. For example, livestock and wildlife can be exposed to PFAS through contaminated water, soil, substrate, air, or food. In this review, we have analyzed and exposed the characteristics of PFAS and their various uses and reported data on their presence in the environment, from industrialized to less populated areas. In several areas of the planet, even in areas far from large population centers, the presence of PFAS was confirmed, both in marine and terrestrial animals (organisms). Among the most common PFAS identified are undoubtedly perfluorooctanesulfonate (PFOS) and perfluorooctanoic acid (PFOA), two of the most widely used and, to date, among the most studied in terms of toxicokinetics and toxicodynamics. The objective of this review is to provide insights into the toxic potential of PFAS, their exposure, and related mechanisms.

## 1. Introduction

Per- and polyfluoroalkyl substances (PFAS) are the active ingredients of fluoro surfactants. PFAS, CnF2n+1−R, refer to a family of chemicals that have been produced since the late 1940s. Fluorine, due to its electronegativity and small size, is responsible for those properties, such as higher acidity, water repellency, and oleophobicity, that make the perfluoroalkyl (CnF2n+1−) fraction better for many uses than its hydrocarbon counterparts. To date, the PFAS structure is used for a wide variety of industrial and consumer applications, such as cosmetics, fire-fighting foams, mining, household products, clothing, pesticides, or medical devices.

Since the late 1990s, the scientific and regulatory community and the public have focused attention on long-chain PFAS and their precursors, such as perfluoroalkanesulfonic acid (PFSA) and perfluoroalkyl carboxylic acid (PFCA), perfluorooctanesulfonate (PFOS), and perfluorooctanoic acid (PFOA). Over the years, PFAS production has continued, with a shift in production toward short-chain PFAS, leading to more frequent measurements of these chemicals in the environment, some of which also appear to be persistent for a long time [1]. Due to their widespread use, PFAS have entered our environment as emerging contaminants and their monitoring is thus vital to facilitate environmental management and remediation. The ability to persist over time leads PFAS to accumulate to trace levels in different environmental compartments, one of the main problems limiting their analysis. Since they are not naturally produced molecules, apart from rare exceptions such as monofluorinated compounds produced by plants or perfluoroalkylates originating from geophysical processes, but all in negligible quantities, their presence in the environment is exclusively of anthropogenic origin [2,3].

### 1.1. Presence of PFAS Worldwide

Epidemiologists and toxicologists around the world have labeled PFAS as “forever chemicals” precisely because of their ability to persist over time in the hydrosphere. Several studies have shown that PFAS once in the body have a rather long half-life, preferentially accumulating in the blood and liver and can cause hepatoxicity, immunotoxicity, neurotoxicity, hormonal alterations in reproduction and development [4,5]. Contamination of water, and subsequent contamination of Food of animal and plant origin are the main contributors to PFAS toxicity to humans, being able to tap into the various sources where they accumulate environmentally [1,6]. This poses a huge risk factor for the health both animals than human, as often what happens in the marine and terrestrial animals anticipates what will happen in humans. Ecological studies conducted on different geographical areas, such as Europe, Asia, North America, or remote polar regions have shown the presence of PFOS in the tissues of different animals, such as polar bears, river otters, bald eagles, dolphins, penguins, seals, Arctic and Antarctic [7,8,9,10]. Levels of PFAS were also found in minor organisms, such as aquatic insect larvae, emerging aquatic insects, at two sites near a point source of PFAS. Considering that aquatic insects may account for 50–90% of the monthly energy balance of terrestrial insectivorous organisms such as birds, this strengthens the hypothesis that minor organisms close contact with polluted waters are the means of quantitative PFAS transfer from water to land [11]. Wild animals from different areas presented different concentrations of PFOS. In fact, it was seen that in the Great Lakes region in North America, the Baltic Sea, and the Mediterranean Sea, wild animals had higher PFOS concentrations than others from remote marine locations, probably because PFAS can bio-magnify when they move from one trophic level to another higher up in the food chain where they can bioaccumulate [7,12,13]. Thus, studies conducted in animal areas have shown a correlation between the presence of PFAS and the industrialization of some areas, and this is also reflected in animal and plant products. In Poland in a study conducted in some industrialized areas, levels of perfluoroheptanoic acid (PFHpA), a widely used PFAS at the industrial level, were observed in honey about 20 percent higher than in no industrialized regions. [14]. 

### 1.2. Regulation and Restrictions on PFAS Use

In 2009, PFOS and PFOA were listed under the Stockholm Convention in Annex A of point 1: Prohibitions and limitations of manufacture and use for commercial products (pesticides and/or industrial chemicals) as Persistent Organic Pollutants due to their demonstrated toxicity, bioaccumulation, persistence in the environment, and ability to travel long distances from the point of release or application [15]. This requires participating countries to eliminate or reduce the release of these chemicals into the environment. The main issues related to PFAS relate to limiting their release into the environment through a restriction of their industrial and commercial use and, consequently, decreasing their presence in living organisms.

Controlling the amounts of PFAS contamination without limiting use is, to date, a dead end; at the same time, we would need other resources to be used as alternatives that do not cause burden once released into the environment. There are several effects of PFAS on living health (domestic and wild animals, humans), and countering their toxicity in a preventive manner may not be easy to date. This review aims to give an overview of the characteristics of PFAS, their use, and the harm associated with their exposure, ultimately reporting some considerations and possible ways to counteract their harmful effects. 

## 2. PFAS Characteristics

### 2.1. Structure and Physical Characteristics

Perfluoroalkyl substances are fully fluorinated (perfluoro-) alkane (carbon-chain) molecules. Their basic chemical structure is a chain of two or more carbon atoms with a charged functional group attached at one end. Polyfluoroalkyl substances are not fully fluorinated. Instead, they have a non-fluorine atom (typically hydrogen or oxygen) attached to at least one, but not all, carbon atoms, while at least two or more of the remaining carbon atoms in the carbon chain are fully fluorinated. Long-chain PFAS are defined as perfluoroalkyl carboxylates (PFCAs) with eight or more carbons, including perfluorooctanoate (PFOA) and perfluoroalkane sulfonates (PFSAs) with perfluorooctane sulfonate (PFOS) with six or more carbons. Short-chain PFAS are defined as PFCAs with seven or fewer carbons, such as perfluorohexanoate (PFHxA), perfluorohexane sulfonate (PFHxS), and PFSAs with five or fewer carbons, such as perfluorobutanesulfonate (PFBS). PFAS are the active ingredients in fluorosurfactants (Table 1). Fluorinated surfactants are used in combination with hydrocarbon foaming agents to produce aqueous film-forming foam (AFFF) used against high-risk flammable liquid fires. Their mechanism involves, when mixed with water, the formation of an aqueous film that spreads over the surface of a hydrocarbon fuel to extinguish the flame and form a vapor barrier between the fuel and atmospheric oxygen to prevent reignition. Fire-fighting foams are divided into Class A and Class B.

### 2.2. Benefits of Using PFAS

**Figure 1:**
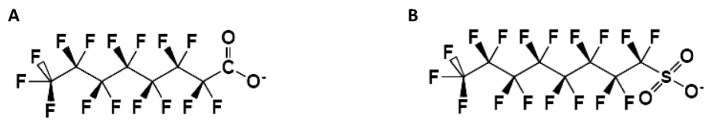
(**A**) Structure of perfluorooctanoic acid (PFOA) and (**B**) perfluorooctanesulfonate (PFOS).

The beginning of PFAS production dates back to the late 1940s, and since then, these molecules have been used for their chemical and physical potential in a wide range of industrial and commercial applications. The earliest ones date back to the 1980s, developed to fight wildfires that broke out in structures. Class B foams are synthetic foams, such as AFFF or alcohol-resistant aqueous film-forming foam (AR-AFFF), and are used to effectively extinguish flammable and combustible liquids and gases, fats, tars, petroleum oils and gasoline, solvents, and alcohols. The chemical structure of PFAS gives remarkable thermal, chemical, and biological stability and inertness to these compounds (Figure 1). The low boiling points and weak surface tension make PFAS extremely resistant to heat and extreme pH values and hardly soluble in either water or lipids. These characteristics result in high resistance to processes of thermal degradation, biodegradation, hydrolysis, and metabolization, resulting in accumulation and persistence in the environment (EFSA, 2008) [16]. In the last decade, the growing interest of the scientific community in these contaminants has led to important discoveries about their potential toxic effect. The amphiphilic nature of these substances prevents their accumulation in adipose tissue, unlike what is usually the case with other halogenated compounds, while they show high affinity for proteins. It is no coincidence, therefore, that PFAS accumulating in the food chain can then also be found in human plasma.

### 2.3. Mechanisms of Action of PFAS

Though extensively studied, the molecular mechanisms of PFAS-induced toxicity are still uncertain. The structural difference of PFAS with other persistent organic pollutants (POPs) makes it unique in toxicity mode. With no aromatic ring, they do not activate the aryl hydrocarbon receptor (AhR) like traditional POPs. With a similar structure to octanoic acid, PFAS is a peroxisome proliferating receptor alpha (PPARα) agonist, making PPARα the most extensively studied signal pathway for PFAS exposure. The PPARs, belonging to the superfamily of nuclear hormone receptors, are ligand-activated transcription factors that play an important role in lipid metabolism. So far, there are three known PPAR subtypes:

PPARα, PPARβ/δ, and PPARγ [17]. Natural ligands that can bind PPARs include various fatty acids as well as numerous fatty acid derivatives and fatty acid compounds [17]. With similar structure to octanoic acid, PFAS is a PPAR agonist. Its PPARα agonist property could explain many toxic effects observed in PFAS -exposed rodents, as well as some epidemiologic evidence in humans, such as the association between the increasing liver enzymes and higher serum PFAS level [18,19,20]. However, many studies also show PPARα-independent mode is also involved in PFAS -induced toxicity. In previous study, it was observed that PFAS -exposed mice had fatty liver, which was not shown in other PPARα- agonist chemicals [21]. Moreover, PFAS -induced liver steatosis was also observed [22]. However, the lipid accumulation contradicted with the activation of PPARα. A potential signal pathway that can substitute PPARα is the activation of other PPARs, i.e., PPARβ/δ and PPARγ. At the structural level, all three receptors have an N-terminal transactivation domain, a highly conserved DNA-binding domain, and a C-terminal ligand-binding domain, the latter of which is deputized of ligand-dependent transactivation. Despite their similarities, each PPAR isoform has unique functions in vivo, probably because of their distinct tissue distributions. Though PPARβ/δ shares similar functions with PPARα, while PPARα is expressed predominantly in the liver, heart and brown adipose tissue, PPARβ/δ is ubiquitously expressed and has a crucial role in fatty acid oxidation in key metabolic tissues such as skeletal muscle, liver and heart [23,24]. PFAS is proved to be both PPARα and CAR agonist, inducing CYP2B10 and CYP4A14 expression in mice. It was demonstrated that CAR and PXR expression was increased in male rats after exposing to Ammonium perfluorooctanoate (APFO) in diet for 28 d [25]. The role of CAR/PXR in PFAS -induced toxicity was further evidenced [26]. In the transcriptome profile of the PPARα-null mice, a large cluster of genes in CAR/PXR signal pathway show expression change upon APFO exposure. This evidence showed that CAR/PXR signal pathway is an important alternative of PPARα signal pathway in PFAS -induced toxicity. Besides PXR/CAR signal pathways, PFAS may also activate other nuclear receptors such as farnesoid X receptor (FXR) and liver X receptor (LXR) due to their similar structure with fatty acids. FXR is an important regulator of bile acid and carbohydrate metabolism [27], while LXR is a particularly important master regulator of lipid and lipoprotein metabolism like PPARα [28]. PFAS treatment could affect some of these nuclear receptors to act as the substitution of PPARα possibly through the cooperation and crosstalk of these NRs [29]. However, in an in vitro cell study, LXR receptor from different organisms including human, mouse, and rat showed no sign of activation by PFAS, which may exclude PFAS from LXR agonist list.

## 3. Commercial Uses

### 3.1. PFAS in Industrial and Consumer Applications

PFAS are widely used commercially, in fact when applied as a coating they impart repellency to moisture and oils/fats by increasing their stability [13]. The chemical and physical capabilities of PFAS make them useful at both industrial and consumer levels, making them a worthy alternative to other less sustainable chemicals [13,30,31]. In addition, PFAS can also be used for resistant coating to moisture, oil, fire, stain and dirt for clothing, upholstery, and carpeting [30]. Industrially, PFAS can be used in metal plating, hydraulic fluids, pesticides, as well as AFFF used as previously discussed [32,33,34]. Overall, most of the commercial use of PFAS today can be classified into four main groups: (1) treatments for durable resistance to moisture and staining in clothing, furs, outdoor equipment, and carpeting; (2) fire-resistant surfactants in fire-fighting foams; (3) chemical-resistant coatings in packaging (including moisture- and oil/grease-resistant food packaging) and goods; and (4) surfactants or precursors in various chemical processes [35].

One important characteristic of PFAS is the production of fluoropolymers, which are widely used, for example, to coat nonstick cookware [36]. They are also used in everyday and household products such as pharmaceuticals, cosmetics, shampoos, household cleaning products, microwave popcorn bags, and floor and automobile waxes [32,37].

### 3.2. Do PFAS Present in Alternative Plastic Packaging Migrate into Food?

Among the various uses of PFAS are paper food packaging coatings, a PFAS surface coating results in an outward orientation of the perfluorinated tail, thus providing water repellency and oleophobicity [13]. In recent years, seemingly less toxic alternatives, such as paper packaging, are being considered to reduce the use of plastics and the associated risks. In fact, with increasing bans on the use of single-use plastics, there has been an increase in biodegradable and compostable paper materials. However, among the various products used in the production of these systems, such as sealing biodegradable paper and tableware, are PFAS. Recently the presence of fluorides, considered indirect evidence of the presence of PFAS, was seen in 42 types of compostable wrappers and containers made of waterproofed paper and plant fiber used in Canadian fast food and restaurants. Among these 42 samples analyzed, the eight samples with the highest fluoride content were selected, and through further analysis, concentrations of PFAS were observed to be three to ten times higher than in the other samples [38]. Among the PFAS found in the analysis, the most represented was found to be 6:2 FTOH (6:2 fluorotelomer alcohol), a compound that, moreover, increases over time being the degradation product of other PFAS. A total of 22 PFAS were identified, from six different chemical groups, in some cases degradation products of other molecules (as in the case of 6:2 FTOH) [38]. Furthermore, a disturbing phenomenon was observed: in dishes left at room temperature for two years, the PFAS concentration decreased up to 85%. This means that, over time, they also disperse in the air and, once they come into contact with food, they can transfer in quantities, especially if stimulated by heat. Furthermore”, underline the authors, “even if they do not migrate towards food, the PFAS of paper and compostable tableware are destined to disperse into the environment, contaminating the air, water, and soil and, in this way, returning to living things through the food chain”. Although 11 US States have already banned the use of PFAS for most food packaging and two major fast food chains plan to ban them by 2025, much remains to be done, and not only in the United States, of course. 

In a second study, conducted by the same research group, focused their attention on a type of plastic not widely used for food but, rather, for cosmetics, pesticides, detergents, and other products: fluorinated high-density polyethylene (HDPE) [39]. Not only does this polymer contain PFAS in concentrations higher than 60 nanograms per gram of plastic but, subjected to various treatments to verify dispersion (for example, in contact with water, methanol, or acetone for a week), it releases quantities between 0, 99 and 66.9 ng/g of plastic. As for food, the quantity that migrates from these containers is lower and between 2.6 and 7.2 ng/g, but splashes at concentrations almost a thousand times higher when heated to 50 °C [39]. Even if HDPE is rarely used in food, there are no laws that prohibit its use, and in any case, according to the authors, materials that so easily disperse large quantities of PFAS should be subject to restrictions and controls, also for limiting environmental contamination [39]. However, there are still not many studies demonstrating the migration of PFAS present in these new packaging systems into food and liquids, but the possibility of their migration already represents a huge risk factor.

### 3.3. PFAS and Pesticides

Among the various applications of PFAS, over the years they have been used as formulation additives to aid pesticide delivery and have been identified as degradation products of pesticide active ingredients [40,41]. In the early years, the Environmental Protection Agency did not mention the presence of PFAS in food pesticides, although it did find the chemicals in non-food plant products [42,43]. Analysis conducted in the U.S. found levels of PFOS in common pesticides, among the most commonly used in several states such as California. In a work conducted from 2020 to 2022, there were discovered PFOS levels several tested insecticides commonly used to treat cotton. Furthermore, it was also detected multiple PFAS species in soil and plant grab samples beyond what was observed in the insecticides tested (PFOS) [41]. All of these findings are part of a dispute between federal regulators and independent researchers over the extent of PFAS contamination in U.S. Pesticides and the related response that eventually led to the stops use of PFAS in Pesticide Products. Following reports and studies on the presence of PFAS in insecticides, some countries have begun monitoring and removing some of the risky products from the market. Registrations for this insecticide have been withdrawn in the United States, but are still allowed in some countries. Although PFAS in pesticide production have been withdrawn and limited in recent years, the problem of their presence and subsequent accumulation in the environment remains high due to their persistence over time. In addition, one must consider the possible synergistic action of pesticides and PFAS in the mechanism of toxicity, both categories being among the most environmentally present. The co-presence of PFAS and pesticides could therefore induce an increased toxic action in long-term exposures as occurs, for example, with co-exposures of other contaminants such as heavy metals, pesticides or endocrine disruptors [44,45,46,47,48].

## 4. PFAS Exposure: The Risks to Animal Health

### 4.1. PFOA and PFOS Toxicology

Most of the experimental studies that have allowed us to understand the long-term effects of PFAS have been conducted on laboratory animals. Among the main consequences of the toxic action of PFAS is the alteration of thyroid functions, but also the ability to induce carcinotoxicity [49]. In a study of chronic PFOS exposure in male Sprague-Dawley rats, researchers found a significant increase in the incidence of thyroid follicular cell adenomas following exposure to 20 ppm PFOS [50]. Other chronic carcinogenicity studies show that PFOA induces benign liver adenomas, Leydig cell adenomas, and pancreatic acinar cell tumors of rat. PFOS also induces liver adenomas in rats [51]. Both PFOS and PFOA have shown moderate acute toxicity after oral or inhalation administration. The lethal dose value (LD50), calculated after single oral administration of PFOS, was 251 and 271 mg/Kg body weight, respectively for female and male rats, with a no observed adverse effect level (NOAEL) of 1 mg/kg/day and a Lowest Observed Adverse Effect Level (LOAEL) of 5 mg/kg/day for developmental toxicity [52]. While for PFOA, an LD50 oral was calculated, within the range of 250–500 mg/Kg body weight for female rats and above 500 mg/Kg body weight for male rats [53]. Inhalation exposure to PFOS in rats, showed a lethal concentration (LC 50) of 5.2 mg/L, with other signs of toxicity such as: slimming, respiratory deficits, and nasal secretions [54]. On the other hand, PFOA was administered via aerosol and after 4 h of inhalation, in male rats, the LC 50 was 980 ng/m^3^ air, and it was observed an increase in liver size and opacity of corneas [55]. Studies in rats and macaques have shown that, in general, prolonged exposure (many weeks) to PFAS, can cause chronic toxic effects, especially in the liver, and determine biochemical alterations associated with lipid metabolism.

### 4.2. PFAS in Domestic Environments and the Risk of Exposure to Pets

Pets like cats and dogs share a common living environment with human beings. Another important consideration is that many diets for dogs and cats are fish-based (especially for cats), so the presence of contaminants in the base product and the consequent accumulation in the pet cannot be excluded. Studying and monitoring the link between contaminants, such as PFAS and pets, does not only provide clear indications on potential risks to their health, but it can also act as a sentinel study on the relationship between human health and contaminants. For example, measuring PFAS in feces can provide information on model of extension and fecal elimination of this class of chemical substances. In fact, there are several studies focused on researching the potential of natural elimination of PFAS occurring within organisms after exposure [56,57,58,59]. A recent study of PFAS elimination by dogs and cats found that longer chain compounds bind to proteins and are excreted at a faster rate than shorter chain PFAS. The authors found that the sum of the concentrations of 13 PFAS ranged from 21.6 to 474 ng/g dry weight for dogs, slightly higher concentrations than those found in cats (range: 18 to 165 ng/g dry weight, average [60]. The daily fecal excretion rates of PFOA, PFNA and PFOS were above the minimal risk levels (MRLs) for intake doses suggested by the Agency for Toxic Substances and Disease Registry’s (ATSDR) for humans, which indicate that pets are exposed to these PFAS at levels above the provisional MRLs [60]. Some sentinel studies have been conducted on domestic cats, also to investigate a possible route of exposure at the PFAS. In a study conducted by Wang et al., on cats in North Carolina, a possible involvement of PFAS with thyroid function was demonstrated. The results showed that circulating concentrations of long-chain perfluorinated carboxylic acids, especially PFNA and PFUnDA, were significantly higher in cat than in humans. Furthermore, serum from hyperthyroid cats showed higher PFAS level (9.50 ng/mL) compared to non-hyperthyroid cats (7.24 ng/mL). In particular, serum PFOS levels were significantly higher in the hyperthyroid cats. This result may indicate a possible link between PFAS levels and hyperthyroidism in cats [61]. However, studies on PFAS exposure for pets are few and it is not easy to understand their long-term toxic effects, also on reproduction and offspring.

### 4.3. PFAS Exposure and the Health Management of Farm Animals

Animals most exposed and affected by PFAS toxicity include livestock and game species. Sources of PFAS exposure are multiple for livestock and game, both environmentally and in relation to commercial products. Studies have shown how feed or compostable packaging used for the feed itself can contain traces of PFAS that can then be transmitted to the animal [62]. Moreover, farm animals often draw water, like domestic animals, are in contaminated sites, or the increase in forest and forest fires and the use of foams and retardants increase the risk factors for this category of animals [63,64].

Studies on PFAS in livestock and game species to date are not many, which complicates farm animal health management and food safety regulation. The major PFAS found in livestock and game studies are PFOS, PFHxS, and PFOA. Concentrations in livestock of environmental PFAS were evaluated in some studies. Substantial differences were found in relation to the areas analyzed, in fact Swedish cattle samples showed lower concentrations than cattle samples taken in Japan [65,66]. In a study of different farm species on collected blood and liver samples, the concentrations of various PFAS were evaluated. PFOS was measurable in all samples and was the most prominent PFAS found in farm animals, with chicken livers containing the highest mean PFOS concentration, followed by livers from pigs and cattle [65]. Similarly, the presence of other PFAS in livestock, such as PFOA, perfluorononanoic acid (PFNA), PFDA, perfluoroundecanoic acid (PFU(nD)A) and perfluorododecanoic acid (PFDoDA), was insignificant [65]. In some livestock studies, forages that were “naturally contaminated,” such as through mixtures of industrial waste with fertilizer, were considered and then used for pilot studies in cattle and sheep [67,68]. The doses of contaminant used, however, were much lower than those reported in toxicokinetic studies on chickens and rats. In most PFAS studies, reported concentrations are not in line with those found environmentally. In fact, livestock studies report tissue concentrations and elimination half-lives after a single bolus dose of PFAS [69,70], in some cases even several months after exposure [71,72]. In farm animals, an important aspect concerns the excretion of PFAS [67]. In a pilot study, conducted in Germany, the excreted levels of PFOS and PFOA in sheep were compared. Two East Friesian sheep were fed for 21 days with contaminated maize, respectively with PFOS 90 μg/kg dry matter, PFOA: 33 μg/kg dry matter. Interestingly, PFOS concentrations were higher in milk than PFOA, and their excretion was also different, with the former being excreted more in the feces and the latter in the urine [67]. In another study, liver PFOS was evaluated in sheep fed with grass obtained from a river floodplain in the Netherlands, with concentrations up to 0.5 μg/kg [73]. Ewes fed contaminated grass for 112 days showed liver PFOS concentrations of 10.9 ng/g *w*/*w*, while animals switched from contaminated to clean grass on day 56 showed a reduction of PFOS in the liver with 9, 2 and 4.7 ng/g *w*/*w* on days 64 and 112, suggesting levels of PFOS intake-dependent accumulation in farm animals [73]. The only livestock studies that reported an approach assessing effects on animal health and tissue concentrations of PFAS were those on poultry. No adverse effects on body weight, organ indexes, blood clinical parameters, or organ histopathology were observed, but pharmacokinetics for PFOA behaved differently than PFOS and PFDA that was similar instead. In fact, it was observed an half-life of 17 days for PFOS, 16 days for PFDA, and 3.9 days for PFOA [74]. Yoo et al., reported a study comparing PFOS and PFOA on male chicken *G. gallus* exposed via subcutaneous osmotic pump for a period of 8 weeks, with exposure during the first four only [75]. No significant differences were found in body index, clinical biochemistry or histology among treatments, except that concentrations of total cholesterol and phospholipids were less in chickens exposed to PFOS, he pharmacokinetic behaviors of PFOA and PFOS are different [75]. Major accumulation sites of PFOA and PFOS were found to be kidney and liver, respectively. PFOA was eliminated much faster than PFOS, with an elimination rate about six fold greater [75].

### 4.4. The Effects of Pre- and Postnatal PFAS Exposure

The risk of environmental contaminants can vary from one species to another, but also depending on the time window of exposure. The harms associated with these substances affect not only those who inevitably exposed, but also and especially future generations, who will inherit the toxicity damage. Every day the gestation of mammals (including women) or other animals, may be exposed to a vast number of environmental chemicals via ingestion, inhalation, or dermal absorption. Tissue partitioning and excretion rates, which determine how and where these chemicals travel through the maternal body, are influenced by physiochemical properties of chemicals as well as maternal and fetal-specific influences. Important chemical properties that play roles in tissue partitioning and/or excretion include charge [76], lipophilicity [77], protein binding affinity [78], solubility [79], and size/length [80]. Maternal and fetal-specific influences include metabolic capabilities [81], as well as parameters that commonly change throughout pregnancy, including tissue volumes and blood flow rates, among others [82]. Exposures during pregnancy include complex mixtures of environmental chemicals, many of which partition into maternal blood and are able to reach the placental barrier through the maternal circulation system [83]. Exposure to PFOS during fetal life was then also evaluated in rats after administration through a feeding tube of increasing dosages from the second to the 21 days of pregnancy. A dose–response relationship that begins to appear between 0.8 and 1.2 mg/kg before becoming statistically significant at 1.6 mg/kg for PFOS administration on dams [84]. It has been shown that the pathological effects are dose-dependent, but in general it has been observed that exposure can compromise postnatal survival, and cause growth and developmental delays and hormonal alterations in survivors [84]. In 2006, evidence of dose-dependent deficits in the growth of mouse infants following oral administration of PFOA to pregnant mothers was reported. Exposure at increasing dosages (1 to 40 mg/Kg body weight per day) was then evaluated [85]. Eye opening delay and growth retardation were observed in all treated groups (except the one treated with the lowest dose). At intermediate doses, decreased postnatal survival was observed, and embryonic resorption was observed in the group treated with the highest dose [85]. Again, PFOA led to increased liver weight in all mothers [85]. Another study conducted in 2007 by White et al. in pregnant mice sought to determine whether and to what extent the adverse effects of PFOA exposure on infants were related to the gestation period rather than to lactation [86]. The results showed that, in addition to intrauterine exposure, the passage of PFOA through milk in infants may have effects on growth retardation in offspring [86]. No clear clinical signs of PFOS and PFOA neurotoxicity have been reported, but some authors have reported behavioral alterations (particularly hyperactivity), probably related to damage to the cholinergic system [87].

Although the use of PFOS and PFOA has been reduced due to their health impact, the total amount of PFAS introduced into the environment has not been reduced because the long-chain compounds have been replaced by short and ultra-short PFAS. Their biological effects are still relatively unknown, and they are more recalcitrant to clean-up attempts. About the human species, many agencies, such as The Center for Disease Control (CDC), Agency for Toxic Substances and Disease Registry (ATSDR), the US Environmental Protection Agency (EPA), the World Health Organization (WHO), International Agency for Research on Cancer (IARC), and the National Toxicology Program (NTP), have recorded the adverse effects of PFOS and PFOA [54]. 

PFAS are known to be proteinophilic, associating with proteins such as albumin, fatty acid binding proteins, and organic anion transporters [88,89]. Because circulating blood contains many of these aforementioned proteins, PFAS can be detected at high concentrations in maternal blood, allowing for maternal circulation to carry PFAS to the placental interface where transfer into the fetal exposome occurs throughout pregnancy. The strong affinity of PFAS for maternal blood allows these substances to travel to the placenta and into cord blood circulation as is indicated by high correlations between maternal serum and cord blood serum [90,91]. Current data highlight that PFAS with linear structures are usually observed in biological fluids at higher concentrations in comparison to their matching branched isomers [92] and have been identified at higher concentrations in fetal serum relative to maternal serum [93]. This trend may reflect differences in efficiencies between compounds crossing the placenta [94], making PFAS uniquely structured to potentially accumulate in target tissues relevant to the fetal exposome. PFAS concentrations have even been shown to increase in the fetal compartment throughout gestation [95], supporting the need for research to better understand potential implications of exposure on fetal health. Little investigated to date, however, has been the long-term effect that PFAS may cause on different generations following pre-natal exposure. Later-in-life outcomes have been evaluated in relation to PFAS to a limited extent and with mixed results for although it has been established that exposure in the gestational period is a huge risk factor for the unborn, to date the effects on endpoints such as fertility or hormonal dysfunction for those born in subsequent generations are unknown. For example, in a study conducted in Taiwan it was examined the potential associations between PFAS in umbilical cord blood and neurodevelopment at two years of age and identified a reduction in gross motor function associated with PFOS [96]. 

To date, seven epidemiologic studies to date have examined associations between maternal PFAS exposure and child autism spectrum disorder (ASD) [97,98,99]. Although results differed, three studies showed that higher prenatal exposure to different PFAS, like PFOS and PFOA, was associated with increased risk of child ASD [97,100,101]. Potential reasons for inconsistent results among these studies include differences in timing of exposure measures in pregnancy, characteristics of study populations, methods of identification or confirmation of ASD cases, and genetic factors. Other PFAS studies, however, have found no associations between PFAS concentrations in cord blood and a range of outcomes including attention deficit hyperactivity disorder, congenital cryptorchidism, and alterations in endocrine function [102,103]. 

**Figure 2:**
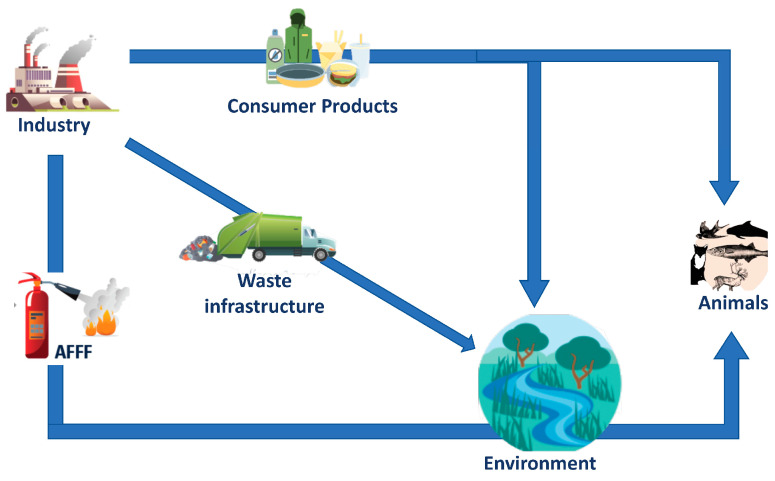
PFAS use pathways, bioaccumulation, and animal exposure. Organisms may be exposed to PFAS via ingestion of contaminated water and food, incidental ingestion of soil and dust, inhalation of contaminated air, and following dermal contact.

It seems almost impossible to limit exposure to PFAS, but even if it could be done, the long-term damage of these substances is already there. Therefore, it becomes necessary to think of an idea to counteract both the use and side effects of PFAS. While industrial and commercial restrictions of these substances should be implemented, unless strictly necessary until a viable less harmful alternative is found. On the other hand, the health effects of those most affected by PFAS toxicity should be countered with preventive action as well as targeted therapies. This can include the action of those natural substances that do not burden the environmental situation as proposed by Green Veterinary Pharmacology (Figure 2).

## 5. Correlation of Geographic Area and Risk

### 5.1. Animals

Monitoring PFAS levels in the animal world, in different geographical areas, is becoming increasingly important not only for their health, but also for human health. Sentinel animals, or those species that share a common environment with humans, are used to measure the extent of exposure when measurement by humans is not practical or possible. The canary in the mineshaft is a classic and probably the best-known example of a sentinel species whose extraordinary sensitivity to toxic gases alerted miners to dangerous occupational conditions. An emerging variation on the theme of animal sentinels relates to an increasing appreciation that animals and humans often share risk to health from exposure to environmental agents. As proposed by the interdisciplinary “One Health” concept, animals may provide more direct information on environmental stressors, food safety, and thus potential risks for human health [104]. As described in the previous paragraphs, some sentinel studies have been conducted on domestic cats, in North Carolina, with high PFAS values (PFNA and PFUnDA), which can be linked to a hyper thyroid condition [61]. Another sentinel study also conducted in North Carolina on dogs and horses showed that average concentrations of total PFAS in horses were lower than in dogs. The dogs in the study had higher concentrations of PFOS, PFHxS and PFOA than the horses, and the researchers highlighted alkaline phosphatase, glucose and globulin in dogs and gamma glutamyl transferase in horses as potential biomarkers associated with PFAS exposure [105]. Through the various pathways of PFAS release into the environment, the potential risk affects not only terrestrial organisms, but also and especially aquatic organisms, water being the major environmental vehicle for these substances. PFAS can be found in very high concentrations in areas with fluorochemical industries. Up to 13.5 μg/L have been detected in surface waters in China [106] and 11 μg/L of PFOA was detected in surface water in Alabama, US [107], while in Europe, Asia and North America often exceed 70 ng/L34 [108]. Correlations between concentrations of several different PFAS in the tissue and health variables like alterations in liver somatic index and body size has been found in Baltic cod [109]. Accumulation of various PFAS has been shown in green mussels (*Perna viridis*), Singapore local species, in screening studies [110,111]. Exposure to 10 μg/L PFOS, PFOA, PFNA and PFDA have shown immunotoxic effects in green mussels [112]. Bioaccumulation of PFASs is a problem not only limited for marine species, but also to freshwater ecosystems. Emerging levels of PFASs such as N-ethyl perfluorooctanesulfonamidoa-acetic acid (NEtFOSAA), bis(perfluorohexyl) phosphinic acid (6:6 PFPiA) have been frequently detected in sediments and/or organisms in Taihu Lake, China [113]. Although PFASs are preferentially used in industrialized and urbanized areas, atmospheric measurements showed that even the most remote areas of the planet, such as the Svalbard Islands, had levels of PFASs in the atmosphere. These atmospheric levels were also reflected in animals in those areas, in fact PFOS, PFNA and PFUnDA levels were found in polar bears in East Greenland, ringed seals in the Canadian Arctic and in arctic foxes in Svalbard [10] (Table 2).

### 5.2. Human

The varied distribution of PFAS in different areas of the globe, depending on urbanization, industrialization, prevention, and limitations, suggests a potential human health risk related to geographic implications. Often a key correlating factor between area contamination and human risk is drinking water. An analysis of PFAS concentrations conducted between 2013 and 2015 in the U.S. states found that major industrial sites that produce or use PFAS, military fire training areas, and wastewater treatment plants are the main contributors to increased PFAS concentrations in public water supplies [120]. However, limited information on geographic differences in serum PFAS concentrations is available. For example in a previous study on female nurses living in inland states had higher concentrations of PFHxS but lower concentrations of PFNA and PFDA than those living in coastal regions [121], but such large regions are too broad to capture locally contaminated areas. Asian women, especially Chinese women, had lower concentrations of PFAS compared with white women. Whereas, both Chinese (Oakland) and Japanese (Los Angeles) women had higher concentrations of PFNA compared with white women at the same site [122]. We were limited in our ability to assess whether differences found between Asian and white women were due to race/ethnicity or geographic location because Chinese and Japanese women were each enrolled in only one site. Within Chinese and Japanese, women who were born outside the US had lower concentrations of most PFAS but higher PFNA concentrations [122]. No differences were seen in PFAS serum concentrations between white women and US-born Chinese women in Oakland suggesting the reason on different lifestyles being maintained after immigration rather than to lower exposure levels before immigration. In Italy, one of the area’s most at risk of PFAS contamination in drinking water is the Veneto region, with significant levels already emerging from studies conducted since 2006 [123,124,125]. A comparison of mortality rates over the period between 1980 and 2013 among residents according to socioeconomic status, habits, and proximity to municipalities with higher PFAS contamination showed differences in mortality but also disease risk. Indeed, the authors showed that residents in the most contaminated areas had higher relative risks for general mortality, diabetes, cerebrovascular disease, cerebrovascular disease, cerebrovascular disease, myocardial infarction, Alzheimer’s disease, and Parkinson’s disease, as well as for kidney and breast cancer and Parkinson’s disease, and kidney and breast cancer [126]. Regardless of the route of PFAS exposure to humans, they pose a serious health concern. Assimilation through contaminated food, either directly or indirectly, e.g. related to packaging methods, poses a huge focus on human health effects. Risk factors associated with PFAS may include developmental, lipid metabolism and endocrine disruption, carcinogenicity, immunotoxicity, hepatotoxicity, and reproductivity. In most human studies, the link between PFAS concentration and lipid status, especially cholesterolemia, has been explored [127,128]. In a past study, some researchers evaluated the link between PFAS and cholesterol level at the gene expression level [129]. Eriksen et al. (2013) found a substantial positive relationship between PFAS and total cholesterol, a link that also appeared to be influenced by sex and the presence or absence of diabetes [127]. In other studies, links between PFASs and various hormones, such as thyroid [130,131], and sex hormones 245, [131,132], as well as development [132,133] have been explored. The researchers found that only PFOS levels were negatively associated with testosterone, calculated as free testosterone (FT), free androgen index (FAI), and ratios of T/LH, FAI/LH, and FT/LH. Furthermore, after measuring PFAS levels in more than a thousand of patients, both males and females aged 12 to 80 years, there were no significant relationships between PFAS and testosterone, suggested that increases in FT3, TT3, and FT4 among adult females, could be during adolescence, PFAS might be related to increases in TSH among males and decreases in TSH among females [130], suggesting sex-specific effects also in correlation with hormones.

## 6. Natural Substances with Low Environmental Impact

The anthropization, and the widespread presence of environmental pollutants, represent factors that directly affect the environment itself, animal health and, through the food chain, interfere with human health. Therefore, the identification of a new scientific approach, which simultaneously considers the environment’s healthiness, animal well-being and food safety in solidarity with human health, is mandatory. One example of the green conversion is biopesticides, which were created with the very goal of decreasing the use of chemicals that are dangerous if accumulated in the environment. According to the World Health Organization (WHO), pesticides are a major threat to the environment and humans [134], and this is even more important because of PFAS co-contamination. For this reason, new pesticides with novel natural and synthetic components are developed, which are less toxic, specific to a target pest, effective in small quantities, and decompose more quickly than conventional pesticides [135]. Biopesticides are natural compounds or agents obtained from animals, plants, and microorganisms such as bacteria, cyanobacteria, and microalgae and are used to control agricultural pests and pathogens [136]. The use of biopesticides is far more advantageous than the use of their counterparts, traditional chemical pesticides, because they are environmentally friendly and host-specific [137,138]. The main distinction between biopesticides and synthetic pesticides is their mode of action. Biochemical, microbial, and plant-incorporated protectants (PIPs) are the three types of biopesticides identified by the US Environmental Protection Agency (EPA). Nature offers a high biodiversity of endemic plants with the most diverse nutraceutical functions Part of this knowledge is rooted in the ancient traditions of rural areas and could be reevaluated to scientifically confirm possible therapeutic activity. With a view to environmental and animal protection, the figure of the Green Veterinary Pharmacology and Toxicology, a branch of veterinary pharmacology that aims to be a complementary and sustainable way to reduce the use of chemicals and minimize drug resistance and residue persistence in the environment, from a “One Health” approach perspective. Recently, these approaches have been successfully applied due to their low environmental toxicity, significant antibacterial activity, efficacy in parasite control in small ruminants and in bee farming, providing a valid alternative to conventionally used drugs and whose efficacy is hampered by resistance phenomena. Some work has demonstrated as extracts of the plants *Salix caprea* and *Artemisia campestris* an anthelmintic effect in gastrointestinal nematodes of sheep [139]. Another study demonstrated the potential use efficacy of essential oil of *O. heracleoticum* against *V. destructor*, in the control of varroatosis in honeybee farms [140]. To date, there are numerous studies demonstrating the effectiveness of natural substances in the treatment of diseases. The antioxidant and anti-inflammatory properties of these natural substances allow them to be used both as a long-term treatment and as an adjunct to conventional therapies. Recent studies have demonstrated the action of cashew nuts in several experimental models of colitis, pancreatitis, and hyperhomocysteinemia [141,142,143]. Or even the use of hydroxytyrosol in in vitro models of mastitis by modulating the Nuclear Factor erythroid 2-related transcription factor 2 (NRF2) pathway [144,145].

## 7. Discussion

Although the use of PFOS and PFOA has been reduced due to their health impact, the total amount of PFAS introduced into the environment has not been reduced because the long-chain compounds have been replaced by short and ultra-short PFAS. Their biological effects are still relatively unknown, and they are more recalcitrant to clean-up attempts. About the human species, many agencies, such as The Center for Disease Control (CDC), Agency for Toxic Substances and Disease Registry (ATSDR), the US Environmental Protection Agency (EPA), the World Health Organization (WHO), International Agency for Research on Cancer (IARC), and the National Toxicology Program (NTP), have recorded the adverse effects of PFOS and PFOA [54]. PFAS are known to be proteinophilic, associating with proteins such as albumin, fatty acid binding proteins, and organic anion transporters [88,89]. Because circulating blood contains many of these aforementioned proteins, PFAS can be detected at high concentrations in maternal blood, allowing for maternal circulation to carry PFAS to the placental interface where transfer into the fetal exposome occurs throughout pregnancy. The strong affinity of PFAS for maternal blood allows these substances to travel to the placenta and into cord blood circulation as is indicated by high correlations between maternal serum and cord blood serum [90,91]. Current data highlight that PFAS with linear structures are usually observed in biological fluids at higher concentrations in comparison to their matching branched isomers [92] and have been identified at higher concentrations in fetal serum relative to maternal serum [93]. This trend may reflect differences in efficiencies between compounds crossing the placenta [94], making PFAS uniquely structured to potentially accumulate in target tissues relevant to the fetal exposome. PFAS concentrations have even been shown to increase in the fetal compartment throughout gestation [95], supporting the need for research to better understand potential implications of exposure on fetal health. Little investigated to date, however, has been the long-term effect that PFAS may cause on different generations following pre-natal exposure. Later-in-life outcomes have been evaluated in relation to PFAS to a limited extent and with mixed results for although it has been established that exposure in the gestational period is a huge risk factor for the unborn, to date the effects on endpoints such as fertility or hormonal dysfunction for those born in subsequent generations are unknown. For example, in a study conducted in Taiwan it was examined the potential associations between PFAS in umbilical cord blood and neurodevelopment at two years of age and identified a reduction in gross motor function associated with PFOS [96]. To date, seven epidemiologic studies to date have examined associations between maternal PFAS exposure and child autism spectrum disorder (ASD) [97,98,99]. Although results differed, three studies showed that higher prenatal exposure to different PFAS, like PFOS and PFOA, was associated with increased risk of child ASD [97,100,101]. Potential reasons for inconsistent results among these studies include differences in timing of exposure measures in pregnancy, characteristics of study populations, methods of identification or confirmation of ASD cases, and genetic factors. Other PFAS studies, however, have found no associations between PFAS concentrations in cord blood and a range of outcomes including attention deficit hyperactivity disorder, congenital cryptorchidism, and alterations in endocrine function [102,103]. 

It seems almost impossible to limit exposure to PFAS, but even if it could be done, the long-term damage of these substances is already there. Therefore, it becomes necessary to think of an idea to counteract both the use and side effects of PFAS. While industrial and commercial restrictions of these substances should be implemented, unless strictly necessary until a viable less harmful alternative is found. On the other hand, the health effects of those most affected by PFAS toxicity should be countered with preventive action as well as targeted therapies. This can include the action of those natural substances that do not burden the environmental situation as proposed by Green Veterinary Pharmacology.

## 8. Conclusions

In conclusion, in this review we provide an overview of PFAS, from their varied uses due to chemical/physical characteristics to their harm to animal health. As we are previous cited, what happens in the animal is often no more than an anticipation of what may happen in humans, and animal studies can help provide insight into the effects of PFAS exposure in a chronically exposed population. To date, animals are the most targeted by the PFAS threat. In fact, the sources to which animals are exposed are multiple and this would require more screening in products placed on the market. Current knowledge is insufficient to address the hazard associated with these chemicals, both in terms of toxicokinetics and biodistribution. In addition, the main gaps identified in the current literature are the lack or limitedness of studies investigating the route of exposure, as most of them have not been defined, the critical time window of exposure, a mixture of PFAS, and various dose-response relationships between PFAS and target endpoints. Considering the current limits of knowledge on the effects of PFAS in animals, this review is intended to be a point of reflection on the effects, limitations, and options to counter them.

## Figures and Tables

**Table 1:** Classification of Per- and polyfluoroalkyl substances (PFAS).

PFAS Group	PFAS Acronym	PFAS Name
Short-chain perfluoroalkyl carboxylic acids (PFCAs) (With less than seven carbons)	PFHpA	Perfluoro-n-heptanoic acid
	PFHxA	Undecafluorohexanoic acid
	PFPeA	Perfluorovaleric acid
	PFBA	Perfluorobutanoic acid
Short-chain perfluoroalkyl sulfonic acids (PFSAs) (With less than five carbons)	PFBS	Perfluorobutanesulfonic acid
	PFPeS	Perfluoropentanesulfonic acid
Long-chain perfluoroalkyl carboxylic acids (PFCAs) (With more than seven carbons)	PFOA	Perfluorooctanoic acid
	PFNA	Perfluorononan-1-oic acid
	PFDA	Nonadecafluorodecanoic acid
	PFUnDA	Henicosafluoroundecanoic acid
	PFDoDA	Tricosafluorododecanoic acid
Long-chain perfluoroalkyl sulfonic acids (PFSAs) (With more than five carbons)	PFOS	Perfluorooctane sulfonate
	PFNS	Perfluorononanesulfonic acid
	PFDS	Perfluorodecanesulfonic acid

**Table 2:** PFAS in wild, farm, and companion animal studies.

Animals	PFAS Acronym	PFAS Concentrations	Location	Studies
Cats	PFNA, PFUnDA, PFHxS	18.0–165 ng/g dw	North Carolina USA	[60,114]
Dogs	PFOA, PFNA, PFOS, PFHxS	21.6–474 ng/g dw	North Carolina USA	[60,105,114]
Horses	PFOA, PFOS,	0.10 ng/mL 1.8 ng/mL	North Carolina USA	[105]
Chickens	PFOA, PFOS, PFHxS	4.75, ng/g ww 25.7 ng/g ww 4.29 ng/g ww	China	[115]
Barnacle geese	PFOS	1.21 ± 2.97 ng/g	Norway, UK	[116]
White-tailed eagle nestlings	PFOS	4.58 e 52.94 ng/mL	Norway, UK	[117]
Polar bear	PFOS, PFNA, PFUnDA	205 ng/mL 37.6 ng/mL 25.5 ng/mL	Greenland, Norwegian Arctic	[10]
Cattle	PFOS,	3.0 ng/ml	Sweden, Japan	[65,66]
Sheep	PFOS	103 and 240 μg/L (Pilot study after exposure to 1.16–1.45 μg/kg bw/d)	Germany, Netherlands	[67]
Whales	PFNA, PFDA PFUnD	50 ng g^−1^ ww	Faroe Islands	[118]
Carps	PFOS, PFHxS	1.43 nmol/gw 5.20 nmol/gw	China	[119]
Aquatic organisms	PFHxS, PFOA	0.141–74.9 ng/g ww	China	[113]

PFOA (perfluorooctanoic acid); PFOS (perfluorooctane sulfonate), PFNA (perfluorononanoic acid); PFHxS (perfluorohexane sulfonate); PFUnDA (Perfluoroundecanoic acid).
